# Vitamin D Deficiency and Markers of Liver Disease in American Indian Adolescents: The Strong Heart Family Study (SHFS)

**DOI:** 10.3390/biomedicines14061277

**Published:** 2026-06-03

**Authors:** Jessica A. Reese, Erin Davis, Pryce Holloway, Amanda M. Fretts, Tauqeer Ali, Elisa T. Lee, Jason G. Umans, Jason F. Deen, Michael S. Middleton, Rohit Loomba, Claude B. Sirlin, Shelley A. Cole, Ying Zhang, Jennifer D. Peck

**Affiliations:** 1 Center for American Indian Health Research, Hudson College of Public Health, University of Oklahoma Health Campus, Oklahoma City, OK 73104, USA; jessica-reese@ou.edu (J.A.R.); tauqeer-ali@ou.edu (T.A.);; 2 Department of Biostatistics and Epidemiology, Hudson College of Public Health, University of Oklahoma Health Campus, Oklahoma City, OK 73104, USA; 3 Department of Anatomical Sciences and Neurobiology, University of Louisville, Louisville, KY 40202, USA; 4 Department of Epidemiology, University of Washington School of Public Health, Seattle, WA 98195, USA; amfretts@uw.edu; 5 MedStar Health Research Institute, Washington, DC 20010, USA; 6 Georgetown-Howard Universities Center for Clinical and Translational Science, Washington, DC 20007, USA; 7 Departments of Pediatrics and Medicine, University of Washington, Seattle, WA 98195, USA; 8 Liver Imaging Group, Department of Radiology, University of California at San Diego, La Jolla, CA 92093, USA; 9 Texas Biomedical Research Institute, San Antonio, TX 78245, USA

**Keywords:** prospective studies, vitamin D deficiency, liver diseases, adolescent, American Indian

## Abstract

**Background:** Vitamin D deficiency (VDD) may be associated with chronic health issues, including markers of liver disease; however, evidence in American Indian adolescents is limited. Therefore, we aimed to evaluate the relationship between VDD in American Indian adolescents and markers of liver disease, including both serum and magnetic resonance imaging (MRI) markers. **Methods:** The Strong Heart Family Study (SHFS) is a multicenter, family-based, prospective cohort study among American Indians. We evaluated SHFS participants who were <20 years old at baseline (2001–2003; n = 308), defined VDD as hydroxyvitamin-D (25[OH]D) ≤ 20 ng/mL and calculated the Hepatic Steatosis Index (HSI). In 2006–2009, we measured follow-up serum markers (n = 269, median follow-up = 5.8 years, range = 3.0–8.5), and in 2018–2020, we collected MRI markers of hepatic steatosis and fibrosis (n = 33, median follow-up = 16.7 years, range = 15.3–18.1). **Results:** At baseline, a greater proportion of those with VDD had HSI > 36 (64.5% with VDD; *p* = 0.002). For participants who reported consuming alcohol, serum alkaline phosphate (ALP) increased at follow-up, which was higher for those with versus without VDD (beta = 12.24, 95% CI = 1.23–23.24). For participants who reported not consuming alcohol, ALP decreased but was not different between the VDD groups. The MRI-proton density fat fraction was higher in those with (median = 11.0%, IQR = 7.7–19.2%) versus without (median = 4.4, IQR = 1.8–6.3%) VDD at baseline (*p* < 0.001). **Conclusions:** VDD may be associated with markers of liver disease in American Indian adolescents. After 5.5 years, VDD was associated with increasing ALP in those who consume alcohol, while controlling for adiposity and other covariates. After 17 years of follow-up, VDD was associated with increased liver MRI-PDFF.

## 1. Introduction

Previously published studies suggest that vitamin D deficiency (VDD) is associated with multiple negative chronic health issues [1]. There is increasing awareness that VDD levels may vary according to ethnicity, due in part to differences in dietary intake and varying levels of absorption through sunlight [2]. Although research on VDD among American Indian adolescents has been limited, a cross-sectional study of American Indian children and adolescents (n = 198) in the Great Plains region reported that elevated 25-hydroxyvitamin D (25[OH]D; <30 ng/mL) was nearly universal (97%) and associated with type 2 diabetes mellitus (T2 DM) and with markers of cardiovascular risk, including elevated triglycerides (TG) and hypertension, after adjusting for body mass index (BMI) [3]. Furthermore, a study of cardiovascular risk factors among American Indian adolescents from the Strong Heart Family Study (SHFS) reported the following: (1) participants with metabolic syndrome were more likely to have VDD; and (2) participants with VDD at baseline were more likely to develop T2 DM during 13 yrs of follow-up, compared to participants without VDD [1]. Due to the potential for negative health outcomes as early as adolescence, it is important to recognize VDD early and identify potential opportunities for intervention [1].

Metabolic Dysfunction-Associated Steatoic Liver Disease (MASLD) is a condition characterized by the accumulation of fat in hepatocytes, and it can progress to more severe forms of liver disease [4]. MASLD is estimated to affect 25% of the population worldwide, with the prevalence of MASLD in the pediatric population estimated to be 13% (estimates range from 1% at age 2–4 yrs to 17% in adolescents) [4]. It is thought that vitamin D may contribute to adipose tissue regulation and may be involved in MASLD pathogenesis [5]. Studies have reported inverse relationships between vitamin D levels and the risk of developing MASLD [6,7], although results from observational studies are conflicting [8]. The relationship between VDD and MASLD in adolescence is of particular interest given the high prevalence of MASLD, but evidence is inconsistent due to the limitations of cross-sectional studies and lack of control for adiposity [9].

The SHFS is a prospective cohort study of cardiovascular disease in American Indian populations [10]. The SHFS provides an opportunity to evaluate the relationship between VDD and markers of liver disease among American Indian adolescents using both cross-sectional and prospective assessments, while controlling for adiposity [10]. Therefore, we aimed to evaluate associations between VDD and serum markers of liver disease, alanine aminotransferase (ALT), aspartate aminotransferase (AST), AST/ALT, alkaline phosphate (ALP), bilirubin, the Hepatic Steatosis Index (HSI) [11], and the fibrosis score (FS) [12] among American Indian adolescents. We also describe associations between VDD in adolescence and MRI measures of steatosis [13], fibrosis [14], and a serum marker of liver necrosis in adulthood [15].

## 2. Material and Methods

Study Population: The SHFS is a multicenter, prospective cohort study that includes 12 American Indian communities and tribes living in Arizona, Oklahoma, and the Dakotas [10]. We included adolescents who participated in the SHFS baseline examination (2001–2003) with measurements of vitamin D [1]. In addition, we invited all baseline participants to take part in the first follow-up examination (2006–2009, median follow-up time = 5.8, range = 3.0–8.5 years). A selection of baseline participants took part in the SHFS liver study, a second follow-up examination (2018–2020, median follow-up time = 16.7, range = 15.3–18.1 years). Recruitment for the SHFS liver study was stratified equally by obesity (BMI ≥ 30 kg/m^2^), type 2 diabetes (T2 DM, fasting glucose ≥ 126 mg/dL or taking diabetes medication), and self-reported heavy episodic alcohol consumption (consuming ≥ 5 alcoholic drinks on the same occasion in the past 30 days). The protocols and procedures were approved by participating tribal communities, the institutional review boards of Indian Health Service (IRB #: P-06-01-OK, approval date: 5 March 2019) and the participating institutions (University of Oklahoma Health Campus IRB #: 10188, approval date: 11 January 2019). We obtained informed consent from every participant. This research was conducted in accordance with both the Declarations of Helsinki and Istanbul.

25[OH]D Assessment: Trained study personnel collected serum using standardized protocols. Similarly, methods of vitamin D collection and measurement were standardized and have been previously described [1]. We defined VDD according to the Institute of Medicine recommended serum cut points for 25[OH]D: deficient ≤ 20 ng/mL (≤50 nmol/L) and sufficient > 20 ng/mL (>50 nmol/L) [16].

Markers of Liver Disease Assessment: Serum markers of liver disease (ALT, AST, AST/ALT ratio, ALP, and bilirubin) were assayed using a Vitros.5.1 analyzer at both the baseline and the first follow-up visit [17]. We used information from baseline to determine cross-sectional associations with VDD and measures from the first follow-up to determine change in markers of liver disease. We also calculated HSI, using AST, ALT, BMI, sex, and the presence of T2 DM, with HSI ≥ 36 indicating likely presence of hepatic steatosis [11]. To determine fibrosis, we calculated an FS based on age, BMI, the presence of impaired fasting glucose or T2 DM, AST/ALT ratio, platelet count, and albumin, with levels ≥ −1.455 indicating likely hepatic fibrosis [12]. In addition, we used information collected in the SHFS liver disease ancillary study to describe MRI markers of steatosis and fibrosis. We included MRI-proton density fat fraction (PDFF), which is a continuous, quantitative imaging marker of hepatic steatosis [13], with elevated hepatic steatosis defined as MRI-PDFF ≥ 5% and normal as PDFF < 5% [13]. Magnetic Resonance Elastography (MRE) is a continuous quantitative marker of liver stiffness, which correlates well with histologic fibrosis. Any fibrosis was defined as MRE liver stiffness ≥ 2.88 kPa (histologic fibrosis stages F1 through F4) compared to no fibrosis defined as MRE liver stiffness < 2.88 kPa (histologic fibrosis stage F0) [14]. Finally, serum ferritin was included as an indirect measure of liver necrosis, with elevated ferritin defined as ≥300 ng/mL and normal ferritin defined as <300 ng/mL [15].

Covariate Assessment: We selected several covariates, which investigators have previously reported to be associated with both VDD and markers of liver disease [1]. During the baseline and first follow-up examinations, we collected self-reported demographic and clinical characteristics (age, sex, current smoking, and alcohol consumption) [18]. To determine the amount of vitamin D intake at baseline, we administered the Block Food Frequency Questionnaire (FFQ). In addition to the questions on the standard Block FFQ, we included supplemental questions about consumption of common American Indian foods, such as menudo, pozole, guava, red or green chili, Indian taco, fry bread, corn tortilla, flour tortilla, and spam [19].

Since all the participants were adolescents at baseline, we defined obesity as the 95th percentile of BMI based on age, developed by the National Center for Health Statistics [20]. Similarly, we defined increased waist circumference (WC) based on age- and sex-specific cutoffs for adolescents [21]. We directly measured hypertension, T2 DM, lipids, kidney function, and metabolic syndrome, which have been previously described [1]. For this analysis, we defined dyslipidemia as any of the following: (1) total cholesterol (TC) ≥ 200 mg/dL, (2) low-density lipoprotein cholesterol (LDL-C) ≥ 100 mg/dL, (3) high-density lipoprotein cholesterol (HDL-C) < 40 mg/dL for men or <50 mg/dL for women, (4) TG ≥ 150 mg/dL, or (5) taking lipid medication [22]. Finally, we defined metabolic syndrome when at least three of the five components for the syndrome were present, based on age and gender specific cut points for the following: (1) increased WC, (2) elevated blood pressure, (3) elevated TG, (4) elevated fasting glucose, or (5) low HDL-C [1,23].

Statistical Analysis: We used descriptive statistics to summarize the demographic and clinical characteristics of the total study population and stratified by baseline VDD (≤20 versus >20 ng/mL) [16]. Next, we calculated the mean change in markers of liver disease (AST, ALT, AST/ALT, ALP, bilirubin, HSI, and FS) for the total study population and stratified by baseline VDD. Mean change was calculated by subtracting baseline from first follow-up values; therefore, positive values indicate an increase, and negative values indicate a decrease. To determine if the characteristics were different between the VDD groups, we used generalized estimating equations (GEEs) to estimate logistic models while accounting for familial clustering. To address multiplicity across outcomes, we used the Benjamini–Hochberg False Discovery Rate correction.

We used linear mixed models to calculate beta estimates and 95% confidence intervals (CIs) to assess the multivariable relationship between VDD and the change in markers of liver disease while adjusting for covariates and accounting for family clustering. Each association met the assumptions of linear mixed models. Initially, we calculated the univariate association; then, we included age, sex, and study center (Arizona, Oklahoma, or the Dakotas). In the full model, we added the BMI ≥ 95th percentile, hypertension, T2 DM, dyslipidemia, current smoking, current alcohol consumption, and season of blood collection (fall/winter vs. spring/summer). Since VDD may vary with sunlight exposure [24], we used study center and season of blood collection as surrogates for sunlight exposure. We evaluated interaction between VDD and each covariate by including appropriate two-way interaction terms in the models.

Similar to the full study sample, for the 33 participants who had liver MRIs, we summarized the baseline characteristics (age, sex, study center, BMI ≥ 95th percentile, hypertension, dyslipidemia, metabolic syndrome, current smoking, and current alcohol consumption) for the total sample and stratified by VDD status at baseline. Similarly, we described hepatic steatosis (MRI-PDFF as a continuous value, elevated values ≥ 5% PDFF, or normal values < 5% PDFF), liver fibrosis (MRE liver stiffness as a continuous value, any fibrosis (F1 through F4) ≥ 2.88 kPa, or normal values (F0) < 2.88 kPa), and a marker of liver necrosis (serum ferritin as a continuous value, elevated values ≥ 300 ng/mL, or normal values < 300 ng/mL) for the total sample and stratified by VDD. We calculated descriptive statistics, as appropriate, and used GEE linear models to determine differences between the VDD groups. We used a significance level of 0.05 for hypothesis tests and performed statistical analyses in Statistical Analysis System (SAS) version 9.4 (SAS Institute Inc., Cary, NC, USA).

## 3. Results

At baseline, 308 participants met the inclusion criteria of being ≤20 yrs of age and having valid 25[OH]D measurements. Of these, 157 (51%) had VDD and 161 (52%) were female. Compared to the group without VDD, a higher proportion of the VDD group were female (62%; *p* < 0.001), from the Arizona center (80%; *p* < 0.001), had serum collected in the fall/winter (60%; *p* = 0.023), had a BMI ≥ 95th percentile (68%; *p* < 0.001), had dyslipidemia (61%; *p* < 0.001), had metabolic syndrome (65%; *p* = 0.023), and/or were current smokers (62%; *p* = 0.008). Likewise, compared to those without VDD, those with VDD had a higher WC (mean = 37.8, std = 7.2 inches; *p* < 0.001), higher diastolic blood pressure (mean = 70.8, std = 9.3 mmHg; *p* = 0.034), lower vitamin D intake (median = 111.8, IQR = 52.8–207.8 IU; *p* = 0.007) and lower plasma creatinine (mean = 0.7, std = 0.1 mg/dL; *p* < 0.001). Regarding markers of liver disease, those with baseline VDD had lower levels of baseline AST/ALT ratio (mean = 0.8, std = 0.3) compared to those without VDD (mean = 0.9, std = 0.4; *p* = 0.032). Likewise, the overall baseline HSI was higher in those with VDD (mean = 41.9, std = 10.2) compared to those without VDD (mean = 35.9, std = 8.4; *p* < 0.001), with a greater proportion of those with VDD having HSI > 36 (65% vs. 35%; *p* = 0.002), indicating likely hepatic steatosis. All the other markers of liver disease were not statistically different between the VDD groups at baseline (Table 1).

At the first follow-up, an average of 5.75 years later (range = 3.0–8.5 years), 269 (87%) had measurements for markers of liver disease (3 died and 36 were lost to follow-up). During follow-up, AST, ALT, the HSI, and the FS increased, while the AST/ALT ratio, ALP and bilirubin decreased (Table 2). There was a smaller reduction in ALP for those with VDD (mean = −10.9, std = 53.1) compared to those without VDD (mean = −26.0, std =48.4; *p* = 0.022). The mean changes in all the other markers of liver disease were not statistically different between the VDD groups (*p* > 0.05 for all). For the one outcome that was statistically significant (ALP), the corrected alpha is 0.036 (versus 0.05, previously). The observed *p*-value is 0.022, which is lower than the corrected alpha. This indicates that ALP is statistically significant after correction.

When evaluating baseline, multivariable, cross-sectional associations, AST/ALT and bilirubin were associated with VDD in univariate models, but the association attenuated and was not statistically significant when adjusting for covariates (Table 3). However, the association between VDD and ALP was statistically significant when controlling covariates (beta = 14.69, 95% CI = 3.88–25.50). When evaluating two-way interactions between VDD and the covariates, a statistically significant interaction was observed between VDD and current alcohol consumption at baseline, when modeling ALP changes as the outcome. For participants who reported consuming alcohol at baseline, ALP increased at first follow-up, and the increase was higher for those with vs. without VDD (beta = 12.24, 95% CI = 1.23–23.24). For participants who reported that they did not consume alcohol, ALP decreased at follow-up, but the decrease was not statistically different between the VDD groups (beta = −12.58, 95% CI = −33.72–8.56, Table 4).

A median of 16.7 yrs later (range = 15.3–18.1 yrs), 33 (11%) of the baseline participants had liver MRIs and serum ferritin measured during the SHFS liver study, which was considered the second follow-up. At baseline, these 33 participants were an average age of 17.5 years, 21 (64%) were female, and 24 (73%) had VDD. Compared to those who did not have VDD, a greater proportion of those with VDD were female (86%; *p* = 0.020), were from Arizona (89%; *p* = 0.021), had dyslipidemia (92%; *p* < 0.001), and 100% of participants with VDD had BMI ≥ 95th percentile (Table 5). Regarding liver MRI measurements, MRI-PDFF was higher in those with VDD (PDIFF median = 11.0%, IQR = 7.7–19.2%) vs. without (PDFF median = 4.4%, IQR = 1.8–6.3%) at baseline (*p* < 0.001). Likewise, a greater proportion of those with VDD had elevated (≥5%) PDFF (87%; *p* = 0.007). All three participants with advanced fibrosis (MRE ≥2.88 kPa) had VDD at baseline, and the one participant with elevated (≥300 ng/mL) serum ferritin levels, a marker of liver necrosis, had VDD at baseline (Table 6).

## 4. Discussion

This is the first study to evaluate the associations between VDD and markers of liver disease, including both serum and MRI markers, in American Indian adolescents. We report that during adolescence, baseline HSI (which accounts for sex, BMI, and T2 DM diagnosis) was higher in those with vs. without VDD (*p* < 0.001), with a greater proportion of those with VDD having HSI > 36 (indicating likelihood of hepatic steatosis, *p* = 0.002). Five years later, for participants who reported alcohol consumption at baseline, ALP increased, and the increase was higher for those with versus without VDD (beta = 12.24, 95% CI = 1.23–23.24). For participants who reported no alcohol consumption, ALP decreased, but the decrease was not different between the VDD groups. Seventeen years later, MRI-PDFF, a marker of hepatic steatosis, was higher in those with vs. without VDD at baseline. All three of the participants with ‘any fibrosis’ (MRE liver stiffness ≥ 2.88 kPa) and the one participant with elevated serum ferritin, a marker of liver necrosis, had VDD at baseline. These data suggest that vitamin D may play a role in markers of liver disease during adolescence, as well as later in life.

Similar relationships have been reported in other populations. A meta-analysis of eight papers reported associations of low vitamin D levels with MASLD in children and adolescents across the world [7]. Likewise, in a study of 994 adolescents, 14–17 years of age from the West Australian Pregnancy cohort, lower 25[OH]D levels were associated with MASLD, independent of adiposity and insulin resistance [25]. However, the evidence has not been consistent. In a study of 315, mostly Hispanic (87%) overweight and obese children and adolescents, 2–18 years old, VDD was not associated with liver disease defined by imaging and histologic endpoints, which is different than the results presented in this study [9]. However, the study including 315 adolescents may be limited by its cross-sectional design as 25[OH]D and markers of liver disease may vary over time [9]. When evaluating ALT as a marker of liver disease, a study of 3878 Korean adolescents reported that higher levels of ALT (>30 U/L) were associated with VDD independent of obesity and metabolic syndrome [26]. In addition, a larger SHS Liver Study that included adults with a mean age of 50 ± 12 years demonstrated that compared to alcohol consumption, metabolic factors, such as elevated BMI, are stronger risk factors for elevated MRI-PDFF. Furthermore, in our study, about 50% of the adolescents reported consuming alcohol, which is similar to national estimates from the Youth Risk Behavior Survey administered in the year 2000, where 50.1% of participants 12–20 yrs of age reported consuming alcohol in the past 30 days [27].

The underlying causal mechanisms of liver disease remain unclear, but there is some evidence to suggest that obesity and vitamin D play a role [28]. It is thought that the signaling pathway between vitamin D and its receptor through intra-hepatic and extra-hepatic mechanisms might provide insights into the role of vitamin D in liver disease [28]. Potential intra-hepatic mechanisms involve the development and progression of liver disease where vitamin D has been shown in rat models to attenuate inflammation, reduce fibrotic effects, and decrease steatosis [28,29]. Regarding potential extra-hepatic mechanisms, vitamin D deficiency may contribute to liver disease development or progression through effects on adipose tissue biology, including inflammation, insulin sensitivity, lipid metabolism, and adipose tissue remodeling [28,30,31]. In addition, vitamin D may influence liver disease through mechanisms involving the intestinal tissue and gut micro-biota composition [28,32]. Regarding potential mechanisms of the association between VDD and elevated ALP, serum ALP can originate from both hepatic and bone sources. Although ALP is commonly evaluated as part of liver disease assessment, elevated ALP in adolescents may also partly reflect bone-related sources associated with skeletal growth [28,33].

This study was conducted in the largest, prospectively followed cohort of American Indian populations in three regions of the US. Nevertheless, power to determine associations was limited due to smaller numbers of adolescents that participated in the cohort, especially those who underwent MRI examinations (n = 33). Therefore, the results for MRI examinations should be interpreted as preliminary and exploratory. In addition, although we used trained study personnel for serum collection and standardized protocols for measurements of serum vitamin D and markers of liver disease, we cannot rule out residual measurement error. However, we were able observe an association between VDD and HSI, which has been validated in adolescents [34] and those who consume no alcohol or low/moderate levels (≤50 g/day, 98.7% of the study population reported consuming no alcohol or low/moderate levels) [35]. We also report an association between VDD and change in ALP among those with alcohol exposure, while controlling for age, gender, center, BMI ≥ 95th percentile, hypertension, T2 DM, dyslipidemia, current smoking, and season of blood collection. Since the SHFS has three sites across the US, it was designed to be generalizable to American Indian populations. However, the results of this study may not be generalizable to non-American Indian populations, especially populations without similar metabolic risk factors to the study population. In addition, we have one measurement of serum 25[OH]D in adolescence; therefore, we were unable to account for potentially changing serum 25[OH]D levels over time. We used location and season of serum collection as surrogates for sunlight exposure. The prevalence of VDD was higher in participants from Arizona (80% versus 41% in Oklahoma, and 48% in Dakota; *p* < 0.001), which was unexpected because Arizona participants might be likely to receive more sunlight. Therefore, it may be that living in a climate with year-round sunlight is not sufficient to overcome VDD, or that participants may spend more time inside due to high temperatures. More studies are needed to determine the role of sunlight in the association between VDD and markers of liver disease. Since the MRI sub-sample was small (n = 33) and selected through stratified recruitment by obesity, T2 DM, and heavy episodic alcohol consumption, it was not a random sample of the parent cohort. Therefore, MRI findings should be interpreted as exploratory, descriptive, and hypothesis-generating rather than generalizable to the full adolescent cohort. Also, we did not have serial MRI measures; therefore, we were not able to assess longitudinal changes in MRI markers of liver disease over time. Finally, due to these limitations and since this is the first study reporting these associations, the results should not be interpreted as causal.

We demonstrated that VDD in adolescence is associated with adverse markers of liver disease later in life, after controlling for potentially confounding factors and evaluating effect modifiers. Although this observation is consistent with studies in other populations, larger studies with longer follow-up are needed to confirm this observation. In addition, the treatment guidelines released by the Society for Adolescent Health and Medicine indicate that adolescents with serum 25[OH]D levels < 20 ng/mL should be treated with 50,000 IU of vitamin D administered once per week for eight weeks. Then, they suggest repeating the course until levels increase and to supplement with 1000 IU vitamin D daily to prevent recurrence of VDD [36]. Since VDD treatment is recommended, more studies are needed on the effectiveness of vitamin D supplementation at reducing VDD and thereby improving liver health later in life. These could include community health or education programs targeting vitamin D supplementation, with specific emphasis on those with high metabolic risk.

## Figures and Tables

**Table 1:** Comparison of baseline demographic and markers of liver disease in American Indian adolescents who did and did not have vitamin D deficiency (25[OH]D ≤ 20 ng/mL).

Variable at Baseline	Mean (Standard Deviation) or Frequency			*p*-Value
	Total	25[OH]D ≤ 20 ng/mL	25[OH]D > 20 ng/mL	
	n = 308	n = 157	n = 151	
Age (years)	17.4 (1.5)	17.6 (1.5)	17.2 (1.4)	0.050
Sex (female)	161	100	61	<0.001 *
Center				
Arizona	50	40	10	<0.001 *
Oklahoma	101	41	60	
Dakotas	157	76	81	
Vitamin D Intake (IU) ^b^	127.9 (62.4–266.6)	111.8 (52.8–207.8)	142.2 (84.1–349.9)	0.007 *
Vitamin D Supplements (yes)	29	14	15	0.517
Season of collection (Fall/Winter)	85	51	34	0.023 *
BMI ≥ 95th Percentile (yes ^a^)	103	70	33	<0.001 *
Waist circumference (inches)	36.0 (7.0)	37.8 (7.2)	34.0 (6.3)	<0.001 *
Hypertension (yes ^a^)	31	18	13	0.340
Systolic blood pressure (mm Hg)	113.3 (10.8)	113.8 (10.5)	112.8 (11.2)	0.782
Diastolic blood pressure (mm Hg)	69.4 (9.9)	70.8 (9.3)	67.8 (10.3)	0.034 *
Fasting glucose (mg/dL)	91.7 (17.0)	91.7 (15.8)	91.6 (18.2)	0.988
DM Diagnosis (yes)	6	4	2	0.389
Total Cholesterol (mg/dL)	154.5 (30.0)	154.0 (30.6)	155.1 (29.4)	0.367
Low-density lipoprotein cholesterol (mg/dL)	83.0 (24.6)	82.8 (25.6)	83.1 (23.7)	0.543
High-density lipoprotein cholesterol (mg/dL)	49.5 (13.0)	48.0 (12.5)	51.0 (13.4)	0.109
Non-high-density lipoprotein cholesterol (mg/dL)	105.1 (30.9)	106.0 (31.6)	104.1 (30.2)	0.802
Triglycerides (mg/dL) ^b^	93.0 (73.0–132.5)	96.0 (77.0–141.0)	89.0 (70.0–119.0)	0.274
Any Dyslipidemia (yes)	173	105	68	<0.001 *
Plasma Creatinine (mg/dL)	0.8 (0.1)	0.7 (0.1)	0.8 (0.1)	<0.001 *
Metabolic syndrome (yes ^a^)	52	34	18	0.023 *
Current Smoking (yes)	79	49	30	0.008 *
Current Alcohol Consumption (yes)	173	92	81	0.282
AST (IU/L)	24.1 (11.1)	24.5 (12.8)	23.7 (9.0)	0.625
ALT (IU/L)	31.0 (21.1)	33.3 (24.5)	28.6 (16.7)	0.051
AST/ALT ratio	0.9 (0.4)	0.8 (0.3)	0.9 (0.4)	0.032 *
ALP (IU/L)	115.2 (55.1)	112.5 (58.3)	117.9 (51.6)	0.709
Bilirubin (mg/dL)	0.5 (0.3)	0.4 (0.3)	0.5 (0.3)	0.077
Hepatic Steatosis Index	38.9 (9.8)	41.9 (10.2)	35.9 (8.4)	<0.001 *
>36	161	104	57	0.002 *
30–36	83	32	51	0.088
<30	63	21	42	Ref
Fibrosis score	−2.85 (1.5)	−2.83 (1.2)	−2.87 (0.9)	0.529
≥−1.455	23	14	9	0.159
<−1.455	279	142	137	

* Indicates statistical significance, *p*-values are from generalized estimating equations, logistic models, accounting for familial clustering. ^a^ Based on age and sex specific cut points for adolescents. ^b^ Median and interquartile range presented. Hepatic Steatosis Index = 8 × (ALT/AST ratio) + BMI (+2, if female; +2, if diagnosed with T2 DM). Fibrosis score = −1.675 + (0.037 × age) + (0.094 × BMI) + 1.13 (if fasting glucose ≥ 110 mg/dL or diabetic) + (0.99*AST/ALT ratio) − (0.013 × platelet count) − (0.66*albumin).

**Table 2:** Mean changes in markers of liver disease in American Indian adolescents.

Variable	Mean Change (Standard Deviation) ^a^			*p*-Value
	Total	25[OH]D ≤ 20 ng/mL	25[OH]D > 20 ng/mL	
	n = 269 ^b^	n = 133	n = 131	
AST (IU/L)	8.3 (20.2)	10.7 (25.0)	5.9 (13.2)	0.066
ALT (IU/L)	15.9 (29.1)	17.6 (35.1)	14.2 (21.4)	0.433
AST/ALT ratio	−0.1 (0.4)	−0.1 (0.4)	−0.2 (0.4)	0.519
ALP (IU/L)	−18.4 (51.3)	−10.9 (53.1)	−26.0 (48.4)	0.022 *
Bilirubin (mg/dL)	−0.1 (0.3)	−0.05 (0.2)	−0.12 (0.3)	0.052
Hepatic Steatosis Index	6.1 (6.5)	5.5 (5.4)	6.7 (5.8)	0.116
Fibrosis score	0.18 (1.0)	0.11 (1.2)	0.25 (0.9)	0.305

* Indicates statistical significance *p*-values are from generalized estimating equations, logistic models, accounting for familial clustering. ^a^ Mean change was calculated by subtracting baseline from follow-up values. Therefore, positive values indicate an increase, and negative values indicate a decrease. ^b^ 39 either died or were lost to follow-up, median follow-up time = 5.75 yrs (range = 3.00–8.52 yrs).

**Table 3:** Multivariable regression models of the associations between vitamin D deficiency and measures of liver health in American Indian adolescents.

Outcome	Beta Estimate (95% CI) ^a^		
	Univariate	Age, Sex, and Center Models	Full Models ^b^
AST (IU/L)	0.69 (−1.88, 3.25)	0.88 (−1,64, 3.40)	−0.38 (−2.94, 2.18)
ALT (IU/L)	4.17 (−0.68, 9.02)	3.82 (−0.93, 8.57)	0.94 (−3.61, 5.50)
AST/ALT	−0.08 (−0.16, −0.01) *	−0.07 (−0.15, 0.02)	−0.03 (−0.11, 0.05)
ALP (IU/L)	−4.94 (−17.49, 7.61)	15.20 (4.69, 25.72) *	14.91 (3.94, 25.87) *
Bilirubin (mg/dL)	−0.08 (−0.14, −0.01) *	−0.03 (−0.10, 0.03)	−0.02 (−0.09, 0.05)
AST change	4.57 (−0.31, 9.45)	4.71 (−0.43, 9.85)	3.40 (−2.02, 8.82)
ALT change	2.88 (−4.35, 10.11)	6.07 (−1.38, 13.51)	4.48 (−3.34, 12.30)
AST/ALT change	0.03 (−0.07, 0.14)	−0.01 (−0.12, 0.10)	−0.02 (−0.13, 0.08)
Bilirubin change	0.07 (−0.0005, 0.13)	0.05 (−0.02, 0.11)	0.04 (−0.03, 0.12)

* Indicates statistical significance. Median follow-up for the change in markers of liver health was 5.75 yrs (range = 3.00–8.52 yrs). ^a^ Beta estimates from linear mixed models comparing the vitamin D deficiency group defined as 25[OH]D ≤ 20 ng/mL to the reference group, defined as 25[OH]D > 20 ng/mL. All models account for the clustered sampling. ^b^ Models adjusted for age, sex, center, BMI ≥ 95th percentile, hypertension, T2 DM, dyslipidemia, current smoking, current alcohol consumption, and season of blood collection (fall/winter vs. spring/summer).

**Table 4:** Multivariable regression models of the associations between vitamin D deficiency and measures of liver health stratified by alcohol consumption in American Indian adolescents.

Outcome	Beta Estimate (95% CI) ^a^	
	Alcohol Consumption (n = 173)	No Alcohol Consumption (n = 135)
ALP change (IU/L)	12.40 (1.19, 23.61) *	−15.18 (−36.47, 6.10)

* Indicates statistical significance. ^a^ Beta estimates from linear mixed model comparing the vitamin D deficiency group defined as 25[OH]D ≤ 20 ng/mL to the reference group, defined as 25[OH]D > 20 ng/mL. Model accounts for the clustered sampling. Model adjusted for age, sex, center, BMI ≥ 95th percentile, hypertension, T2 DM, dyslipidemia, current smoking, and season of blood collection (fall/winter vs. spring/summer).

**Table 5:** Baseline demographics of the 33 participants that participated in the SHS Liver Study.

Variable at Baseline	Mean (Standard Deviation) or Frequency			*p*-Value
	Total	25[OH]D ≤ 20 ng/mL	25[OH]D > 20 ng/mL	
	n = 33	n = 24	n = 9	
Age (years)	17.5 (1.5)	17.7 (1.6)	17.1 (1.2)	0.096
Sex (female)	21	18	3	0.020 *
Center				
Arizona	18	16	2	0.021 *
Oklahoma	15	8	7	
BMI ≥ 95th percentile (yes ^a^)	20	20	0	NA
Hypertension (yes ^a^)	2	1	1	0.452
Type 2 Diabetes Mellitus (yes)	2	2	0	NA
Any Dyslipidemia (yes)	25	23	2	<0.001 *
Metabolic Syndrome (yes ^a^)	7	7	0	NA
Current Smoking (yes)	6	4	2	0.875
Current Alcohol Consumption (yes)	18	12	6	0.338

* Indicates statistical significance. *p*-values are from generalized estimating equations, logistic models, accounting for familial clustering. ^a^ Based on age and sex specific cut points for adolescents. NA = not able to calculate because of zero cells.

**Table 6:** Description of liver steatosis, fibrosis, and necrosis by vitamin D status at baseline among American Indians participating in the SHS Liver Study.

Markers of Liver Disease	Mean (Standard Deviation) or Frequency			*p*-Value
	Total	25[OH]D ≤ 20 ng/mL	25[OH]D > 20 ng/mL	
	n = 33	n = 24	n = 9	
Liver steatosis measured with MRI				
MRI-PDFF (%, continuous) ^a^	8.5 (4.9–17.5)	11.0 (7.7–19.2)	4.4 (1.8–6.3)	<0.001 *
Elevated MRI-PDFF (≥5% PDFF)	23	20	3	0.007 *
Normal MRI-PDFF (<5% PDFF)	10	4	6	
Liver fibrosis measured with MRI				
MRE-stiffness (kPa, continuous)	2.7 (2.4)	2.9 (2.8)	2.1 (0.5)	0.294
Advanced/mildly advanced fibrosis (≥2.88 kPa)	3	3	0	NA
Normal (<2.88 kPa)	29	21	8	
Liver Necrosis measured with serum ferritin				
Serum ferritin (ng/mL, continuous) ^a^	40.8 (19.3–74.7)	42.3 (16.0–117.6)	38.2 (27.7–57.2)	0.602
Elevated ferritin (≥300 ng/mL)	1	1	0	NA
Normal ferritin (<300 ng/mL)	32	23	9	

* indicates statistical significance. *p*-values are from generalized estimating equations linear models, accounting for familial clustering. ^a^ Median and interquartile range presented. Median follow-up time = 16.7 yrs (range = 15.3–18.1 yrs). NA = not able to calculate.

## Data Availability

All data were collected, analyzed, and reported under agreements made with the sovereign tribal nations that have partnered in this research, which preclude commonly accepted modes of data sharing. Requests to access the dataset from qualified researchers trained in human subject confidentiality protocols may be sent to the Strong Heart Study Coordinating Center at https://strongheartstudy.org/. Requests will be reviewed by tribal research partners before data may be released. This policy is consistent with the “NIH Policy for Data Management and Sharing: Responsible Management and Sharing of American Indian/Alaska Native Participant Data”.

## Abbreviations

25[OH]D	25-hydroxyvitamin D (calcitriol, the major circulating form of vitamin D in the body)
ALT	Alanine Aminotransferase
ALP	Alkaline Phosphate
AST	Aspartate Aminotransferase
BMI	Body Mass Index
CVD	Cardiovascular Disease
CI	Confidence Interval
FS	Fibrosis Score
DBP	Diastolic Blood Pressure
FFQ	Food Frequency Questionnaire
GEE	Generalized Estimating Equations
HIS	Hepatic Steatosis Index
HDL-C	High-Density Lipoprotein Cholesterol
IQR	Interquartile Range
kPa	Kilopascals
LDL-C	Low-Density Lipoprotein Cholesterol
MRE	Magnetic Resonance Elastography
MRI	Magnetic Resonance Imaging
MASLD	Metabolic Dysfunction-Associated Steatotic Liver Disease
PDFF	Proton Density Fat Fraction
SAS	Statistical Analysis Software
SHFS	Strong Heart Family Study
SBP	Systolic Blood Pressure
TC	Total Cholesterol
TG	Triglycerides
T2 DM	Type 2 Diabetes Mellitus
VDD	Vitamin D Deficiency
WC	Waist Circumference
